# Facilitating redo sternotomy: A patented handheld retractor for safe reentry in reoperative cardiac surgery

**DOI:** 10.1016/j.xjtc.2025.07.020

**Published:** 2025-08-27

**Authors:** Rakan I. Nazer, Nabeel A. Ali, Ali M. Albarrati

**Affiliations:** a Department of Cardiac Science, King Fahad Cardiac Center, College of Medicine, King Saud University, Riyadh, Kingdom of Saudi Arabia; b Department of Rehabilitation Science, College of Applied Medical Science, King Saud University, Riyadh, Kingdom of Saudi Arabia

**Figure:**
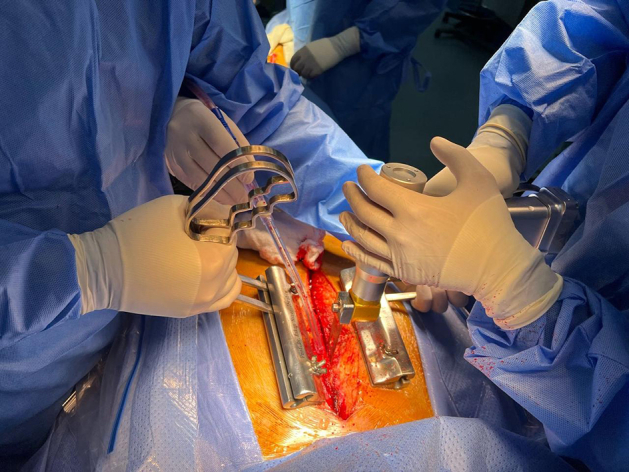
Intraoperative application of the handheld retractor during sternal reentry in redo surgery.

**Central Message**

A handheld retractor enables controlled sternal elevation during redo sternotomy, improving safety and minimizing injury risk in reoperative cardiac surgery (US patent No. US 12, 343, 000 Bl).

Reoperative cardiac surgery is associated with increased morbidity and mortality.^1^ This is partly due to patient comorbidities and complex cardiovascular pathology, but also due to technical challenges such as dense adhesions, lack of anatomic clarity, and the close proximity of vital structures to the posterior surface of the sternum. Critical structures including the innominate vein, right ventricle, and ascending aorta are at risk of injury during sternal reentry and mediastinal dissection.^2^ The increasing number of patients undergoing initial cardiac operations—particularly those with tissue valve replacements, ventricular assist devices, or congenital heart disease—has led to a growing population of long-term survivors who may eventually require redo procedures.

### Technique

All patients underwent a comprehensive preoperative assessment including history, physical examination, evaluation of femoral pulses, and inspection of previous chest incisions. Imaging included echocardiography, posteroanterior and lateral chest radiography, and computed tomography with contrast to assess the proximity of cardiac chambers and vascular structures—such as bypass grafts—to the posterior sternal table. Coronary angiography was performed in patients over 40 years or those scheduled for redo coronary bypass surgery.

Defibrillation pads were placed before induction of anesthesia. For patients not classified as high risk by computed tomography imaging, femoral arterial and venous lines were percutaneously placed. In high-risk cases, a formal femoral cut-down was performed, and peripheral cannulation was established with initiation of cardiopulmonary bypass before sternal reentry.

To prepare for sternal reentry, the previous incision was reopened and dissection carried down to expose the old sternal wires. Each wire was carefully unraveled to leave one free end on each side of the sternum. The sternal midline was marked with electrocautery. Free wire ends were inserted into the jaws of the retractor, which featured knurled inner surfaces and manual screw knobs for secure fixation (Figure 1). Saline lubrication enabled smooth movement of a sliding handle that allowed adjustable traction throughout sawing. If wires were absent or dislodged, new loops of steel wires were carefully passed bilaterally at the chondrosternal junction through the ribs ensuring diligence to stay superficial and not go around the ribs so that the underlying vascular structures are not injured. Dynamic retraction upward stabilized the sternum while counteracting downward pressure from the oscillating saw (Figure 2). The sliding handles on each jaw device allow the dynamic retraction that follows the oscillating bone saw as it works its way from the xiphinsternum at the lower end up to the manubrium at the upper end of the sternum (Video 1).

**Figure 1:**
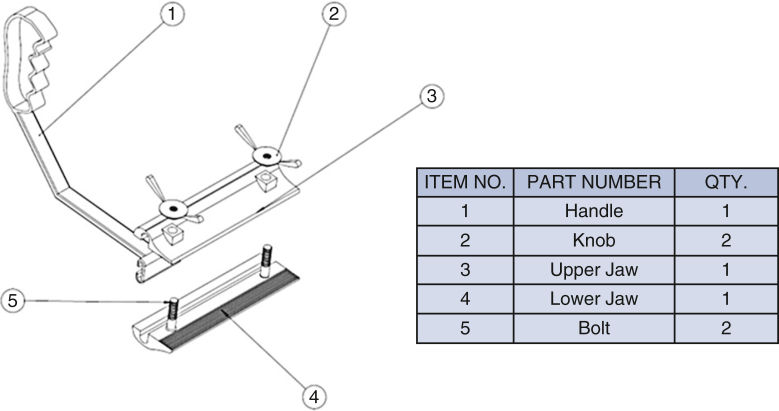
The components and on-table assembly of the retractor tool. Unraveled old sternal wires or newly inserted steel wire loops are fed inside the pressing jaws. The jaws are pressed to tightly hold the free ends of the sternal wires on either side of the sternum by tightening the upper knobs.

**Figure 2:**
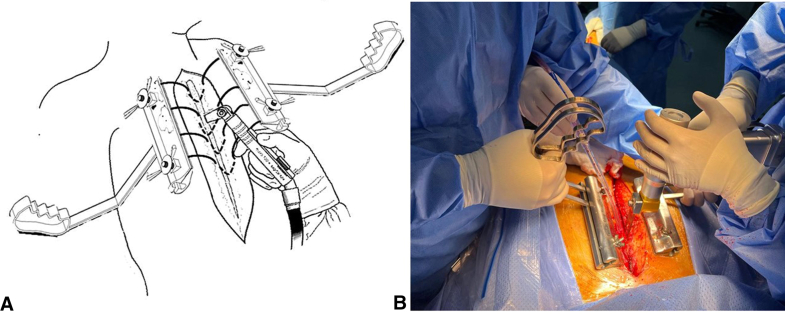
The retractor's application in redo sternotomy. A, Schematic illustration of the utility of the retractors in lifting and stabilizing the sternum during the critical phase of reentry in the reoperative cardiac procedure. B, An actual footage demonstrating the first surgical assistant holding the retractors on either side of the sternum and lifting while the surgeon is applying the oscillating bone saw. The movements of the handles allow for dynamic retraction as the saw is being used on different segments of the sternum.

## Discussion

Although redo cardiac surgery presents a considerable risk, modern techniques have significantly reduced complications. In high-volume centers, reported mortality is as low as 3.5% in 7500 consecutive cases over 10 years.^3^ Key elements for success include team-based approaches, preoperative imaging, careful operative planning, and novel intraoperative adjuncts. Our devised sternal retractor is one such tool that enhances stability and control during sternal division—a critical step in reoperative procedures.

We applied this technique in 40 consecutive redo operations in adult patients. No vascular injury occurred. A survey of 4 senior cardiac surgeons rated the tool highly for usability and safety. All recommended its routine use in appropriate reoperative settings (Online Data Supplement).

## Conclusions

Sternal reentry is often the first critical step in redo cardiac procedures. Thorough evaluation, image guidance, and use of adjunctive tools are essential for patient safety. Exercising good surgical judgment, diligent instrument handling, and proper tissue manipulation remain the cornerstone of preoperative cardiac surgery. Compared with other techniques for stabilizing the sternum in redo surgery with the use of hemostat clamps on each unraveled wire end, the application of a sharp towel clamp, or adapting a modified mammary retractor tool, we believe that the technique described will add another tool in the armamentarium for safety and efficiency.^4^ The handheld sternal retractor described in this report provides dynamic retraction and facilitates a safer, more controlled reentry.

## Conflict of Interest Statement

The authors reported no conflicts of interest.

The *Journal* policy requires editors and reviewers to disclose conflicts of interest and to decline handling or reviewing manuscripts for which they may have a conflict of interest. The editors and reviewers of this article have no conflicts of interest.

## Supplementary Data

Video 1 An intraoperative demonstration of the assembly and application of the sternal retractor in reoperative sternotomy. Video available at: https://www.jtcvs.org/article/S2666-2507(25)00333-5/fulltext.
