# Supplementary figures and images for: Facilitating redo sternotomy: A patented handheld retractor for safe reentry in reoperative cardiac surgery
Source: JTCVS Tech. 2025 Aug 27;33:136–8. doi: 10.1016/j.xjtc.2025.07.020 (PMC12529703; doi:10.1016/j.xjtc.2025.07.020)

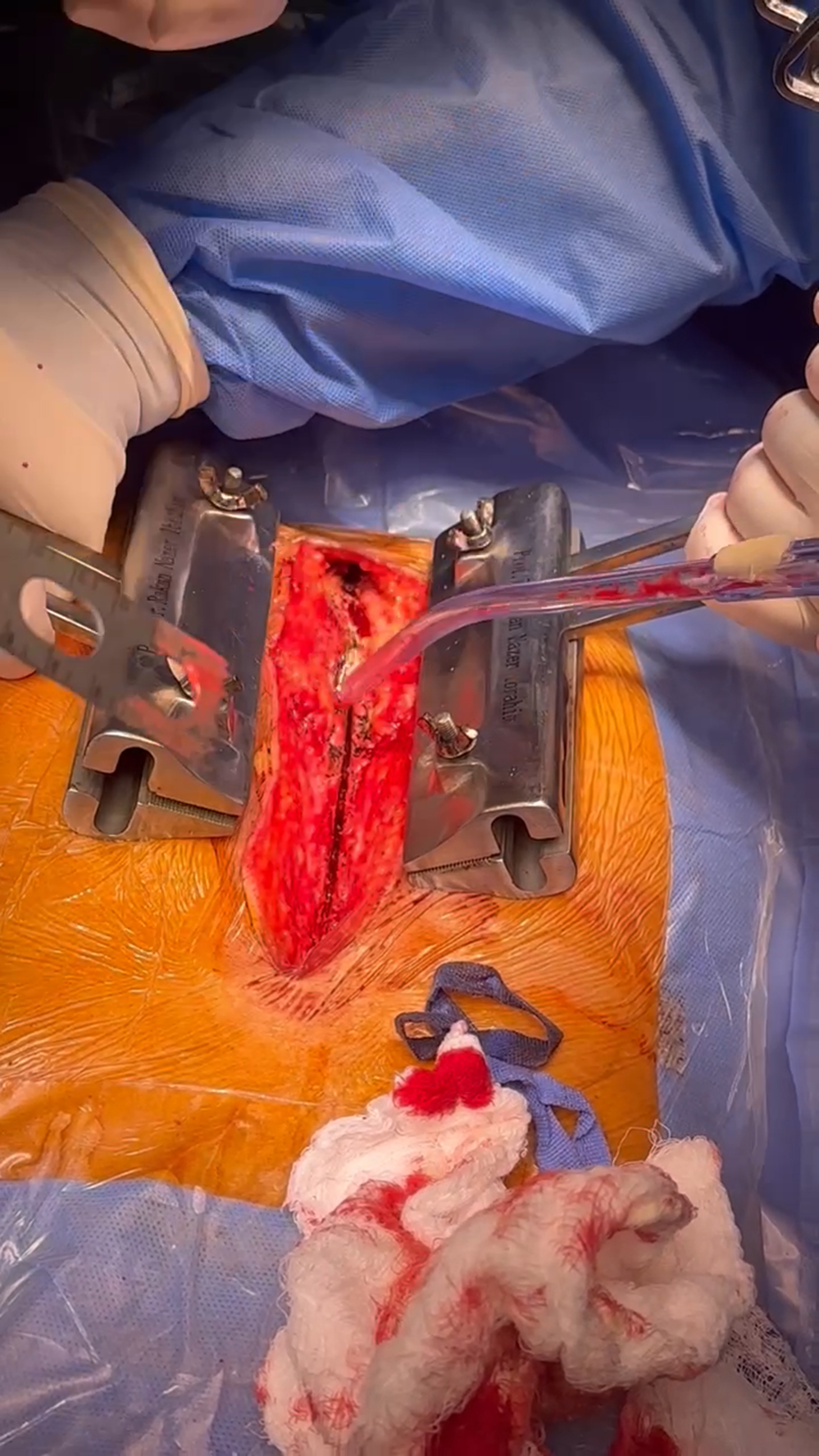

Supplement: Video 1 — An intraoperative demonstration of the assembly and application of the sternal retractor in reoperative sternotomy. Video available at: https://www.jtcvs.org/article/S2666-2507(25)00333-5/fulltext. [file fx2.jpg]
