# Supplementary material for: Facilitating redo sternotomy: A patented handheld retractor for safe reentry in reoperative cardiac surgery
Source: JTCVS Tech. 2025 Aug 27;33:136–8. doi: 10.1016/j.xjtc.2025.07.020 (PMC12529703; doi:10.1016/j.xjtc.2025.07.020)
Supplement: Online Data Supplement [file mmc2.pdf]

Feedbac

Assessment of the utility and use of the Redo-sternotomy Retractor system

1. Was the tool easy to understand

- [ ] Very easy
- [ ] Easy
- [ ] Neither easy nor difficult
- [ ] Difficult
- [ ] Very difficult

2. how did find the assembly of the tool

- ○ Very easy
- ○ Easy
- ○ Neither easy nor difficult
- ○ Difficult
- ○ Very difficult

Assessment of the utility and use of the Redo-sternotomy Retractor system

Feedbac

- Very difficult

3. how was the handling of the tool work during the procedure

- Very satisfied
- Satisfied
- Neither satisfied nor dissatisfied
- Dissatisfied
- Very dissatisfied

4. what was the time of sternal re-entry while using the oscillating sternal saw (in minutes)

0 20

5. Do you think this tool makes redo-sternotomy safe

- Strongly agree
- Agree
- Neither agree nor disagree

Feedbac

0 20

5. Do you think this tool makes redo-sternotomy safe

- Strongly agree
- Agree
- Neither agree nor disagree
- Disagree
- Strongly disagree

6. Do you think this tool saved time compared to using other tool for redo-sternotomy

- Strongly agree
- Agree
- Neither agree nor disagree
- Disagree
- Strongly disagree

◯ Agree

◯ Neither agree nor disagree

◯ Disagree

◯ Strongly disagree

7. Do you recommend having this tool available for your next redo-sternotomy

◯ Always

◯ Usually

◯ Sometimes

◯ Rarely

◯ Never

Done

Powered by
SurveyMonkey®
See how easy it is to create surveys and forms.

Privacy & Cookie Notice

Assessment of the utility and use of the Redo-sternotomy Retracto... system

0

Choose...

Question Summaries | Insights and Data Trends | Individual Responses | Dashboards

Rules | Views | Exports | Shared data | Multi-survey analysis | Share | Save as

RESPONDENTS: 4 of 4

Page 1

Q1

Customize | Export

Was the tool easy to understand

Answered: 4 Skipped: 0

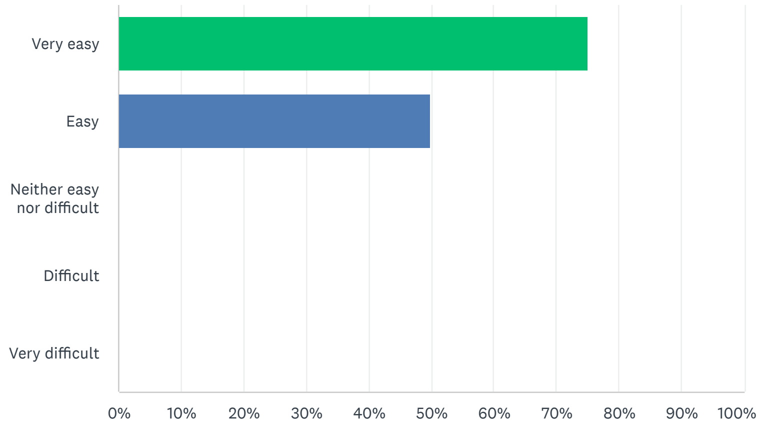

ANSWER CHOICES	RESPONSES	
Very easy	75.00%	3
Easy	50.00%	2
Neither easy nor difficult	0.00%	0
Difficult	0.00%	0
Very difficult	0.00%	0

Total Respondents: 4

Q2

Customize | Export

how did find the assembly of the tool

Answered: 4 Skipped: 0

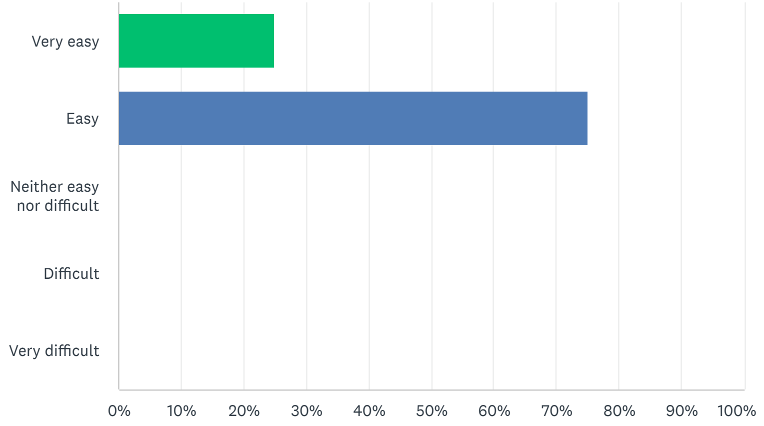

ANSWER CHOICES	RESPONSES	
Very easy	25.00%	1
Easy	75.00%	3
Neither easy nor difficult	0.00%	0
Difficult	0.00%	0
Very difficult	0.00%	0
TOTAL		4

Q3

Customize Export

how was the handling of the tool work during the procedure

Answered: 4 Skipped: 0

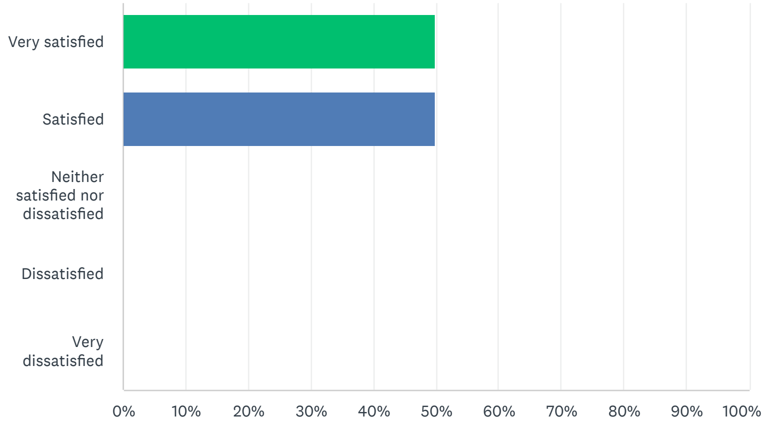

ANSWER CHOICES	RESPONSES	
Very satisfied	50.00%	2
Satisfied	50.00%	2
Neither satisfied nor dissatisfied	0.00%	0
Dissatisfied	0.00%	0
Very dissatisfied	0.00%	0
TOTAL		4

Q4

Export

What was the total time of sternal re-entry while using the oscillating sternal saw (minuets)

Answered: 4 Skipped: 0

RESPONSES (4) WORD CLOUD TAGS (0)

Sentiment Analysis
Detect the sentiment behind written responses to understand how people feel.

Upgrade Watch a demo

Search Responses Filter: by tag

Showing **4** responses

1.3
5/7/2025 06:53 PM View respondent's answers Add tags

4
5/4/2025 05:33 PM View respondent's answers Add tags

3
5/4/2025 05:24 PM View respondent's answers Add tags

3
5/4/2025 03:50 PM View respondent's answers Add tags

Q5

Customize Export

Do you think this tool makes redo-sternotomy safe

Answered: 4 Skipped: 0

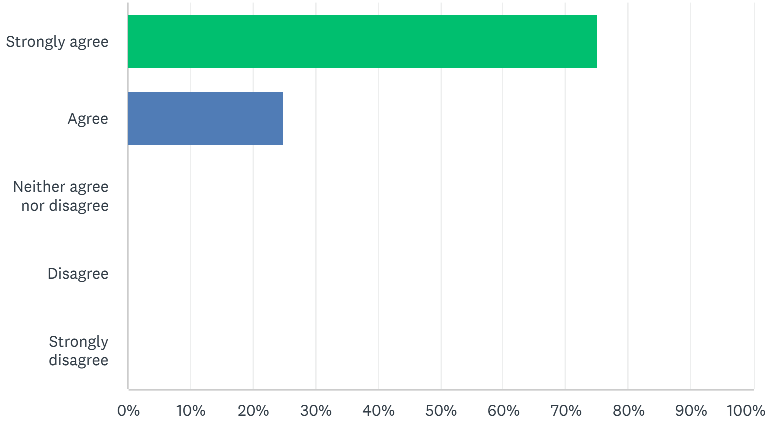

ANSWER CHOICES	RESPONSES	
Strongly agree	75.00%	3
Agree	25.00%	1
Neither agree nor disagree	0.00%	0
Disagree	0.00%	0
Strongly disagree	0.00%	0
TOTAL		4

Q6

Customize Export

Do you think this tool saved time compared to using other tool for redo-sternotomy

Answered: 4 Skipped: 0

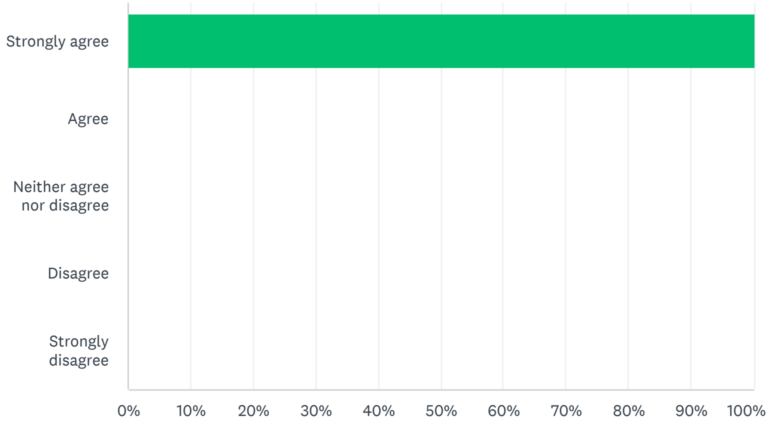

ANSWER CHOICES	RESPONSES	
Strongly agree	100.00%	4
Agree	0.00%	0
Neither agree nor disagree	0.00%	0
Disagree	0.00%	0
Strongly disagree	0.00%	0
TOTAL		4

Q7

Customize Export

Do you recommend having this tool available for your next redo-sternotomy

Answered: 4 Skipped: 0

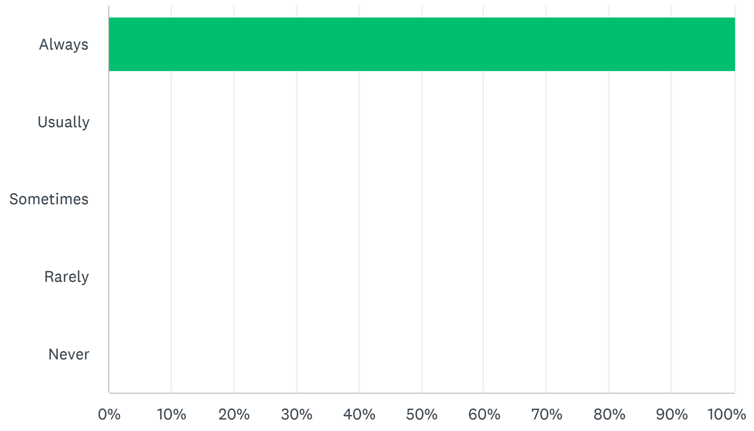

ANSWER CHOICES	RESPONSES	
Always	100.00%	4
Usually	0.00%	0
Sometimes	0.00%	0
Rarely	0.00%	0
Never	0.00%	0
**TOTAL**		**4**

ENGLISH

About SurveyMonkey • Careers • Developers • Privacy Notice • California Privacy Notice • Email Opt-In • Help • Cookies Notice • Copyright © 1999-2025 SurveyMonkey • Customise

TrustedSite CERTIFIED SECURE
